# Role of tenofovir dipivoxil in gut microbiota recovery from HBV-infection induced dysbiosis

**DOI:** 10.1186/s12866-024-03457-4

**Published:** 2024-09-20

**Authors:** Jianfei Long, Maximilian Saw, Pan Zhang, Li Wang, Ling Li, Hongyan Ren, Chao Liu, Zhenxuan Ma, Jiming Zhang, Bin Wang

**Affiliations:** 1 Department of Pharmacy, Huashan Hospital, Fudan University, Shanghai, China; 2 Department of Nephrology, Zhongshan Hospital, Fudan University, Shanghai, China; 3 Department of Pharmacy, Jing’an District Central Hospital, Fudan University, Shanghai, China; 4 Shanghai Mobio Biomedical Technology Co., Shanghai, China; 5 Department of Infectious Diseases, Shanghai Key Laboratory of Infectious Diseases and Biosafety Emergency Response, National Medical Center for Infectious Diseases, Huashan Hospital, Fudan University, Shanghai, China; 6 Department of Infectious Diseases, Jing’An Branch of Huashan Hospital, Fudan University, Shanghai, China

**Keywords:** Hepatitis, Chronic hepatitis B, Gut microbiota, Tenofovir dipivoxil, Dysbiosis

## Abstract

**Background:**

Studies have found dysbiosis of the gut microbiota in individuals infected with the hepatitis B virus (HBV). Tenofovir dipivoxil (TDF) is one of the preferred oral antiviral drugs used for the treatment of chronic hepatitis B (CHB), but the extent to which TDF is able to affect the gut microbiota and inflammatory factors of a patient remains largely unexplored. In this study, we collected stool samples from HBV patients prior to medication and from CHB patients treated with TDF.

**Results:**

The gut microbiota and inflammatory factors were assessed in 42 healthy subjects (HC group), 109 HBV-infected subjects, including 48 CHB patients who were not medicated with nucleoside analogue drugs (No-NAs group), and 61 CHB patients who were medicated with TDF (TDF group). 16 S rRNA sequencing revealed that TDF treatment caused significant changes in the gut microbiota of HBV-infected individuals; however, the gut microbiota of HBV-infected individuals did not fully recover to a pre-dysbiosis state. The relative abundance of *Bacteroidota* gradually decreased from the HC group to the No-NAs and TDF groups. The relative abundance of *Fusobacteriota* was significantly higher in the No-NAs group than in the HC group. At the genus level, *Dialister*, *Eubacterium_hallii_group*, *Halomonas*, *Collinsella*, *Sphingomonas*, *Xanthomonadaceae_unclassified*, and *Rhizobiaceae_unclassified* were overrepresented; while the abundance of *Bacteroides* and *Fusobacterium* decreased significantly in the No-NAs and TDF groups.

**Conclusions:**

This study showed that TDF treatment significantly improved the regulation of the gut microbiota and aided in dysbiosis recovery. We did not observe significant improvement in serum inflammatory factor concentrations, which may be related to the relatively short duration of TDF administration in this study.

**Supplementary Information:**

The online version contains supplementary material available at 10.1186/s12866-024-03457-4.

## Background

Hepatitis B virus (HBV) is a noncytopathic hepatotropic virus. Chronic hepatitis B (CHB) caused by HBV infection is a worldwide epidemic and can lead to severe liver diseases such as liver fibrosis, cirrhosis, and hepatocellular carcinoma (HCC) [1]. The World Health Organization reports that there are approximately 257 million patients infected with chronic HBV worldwide, of whom 887,000 died from complications caused by chronic HBV infection in 2015 [2]. Intestinal bacteria play an important role in maintaining immune system homeostasis and may serve as a barrier to protect the intestinal mucosa against invasion by potential pathogens [3, 4]. Emerging evidence suggests that the gut microbiota may influence the development and progression of liver disease. A shift in the gut microbiome can trigger inflammation, hepatocyte apoptosis, and the progression of liver failure and cirrhosis [5–7]. Viral hepatitis (hepatitis B and C) also has a major impact on the gut microbiota [8], and several studies have reported gut dysbiosis in CHB patients [1, 9]. *Enterococci* are intestinal flora in humans, and it has been shown that patients with CHB and cirrhosis exhibit increased abundance of *Enterococci* [10]. In most liver diseases, intestinal dysbiosis increases the relative abundance of *Proteobacteria*, while decreasing the abundance of *Bacteroidetes* [11]. It has been reported that bacterial translocation occurs in the guts of chronic hepatitis patients [12, 13]. Studies have shown that cirrhotic patients diagnosed with CHB show a decrease in the abundance of *Bifidobacteria* and *Lactobacillus*, while significantly increasing levels of *Enterococcus* [14]. Lu et al. (2011) have suggested that the abundance of *Faecalibacterium prausnitzii*, *Enterococcus faecalis*, *Bifidobacteria*, and lactic acid bacteria differ significantly in the intestines of patients with HBV cirrhosis [12]. Dysregulation of the intestinal microbiota contributes to further exacerbation of HBV infection [15]. Hepatic viruses can disrupt intestinal permeability, lead to intestinal dysbiosis, and release proinflammatory cytokines that contribute to the development of cirrhosis and HCC [16]. In chronic infection with HBV, disruption of the gut microbiota can lead to systemic immune activation [17, 18]. Currently, it is believed that the cause of liver injury is not due to the replication of HBV in hepatocytes, but instead the immune response caused by HBV [18]. Lipopolysaccharide (LPS), also known as endotoxin, is a key component of the outer membrane of Gram-negative bacteria. LPS can secrete many proinflammatory cytokines, including tumor necrosis factor-α (TNF-α), interleukin (IL)-1, and IL -6, which cause liver injury via the NF-κB pathway [19]. In addition, patients infected with HBV have altered intestinal permeability, increasing the endotoxin load in the portal vein, leading to hepatic toll-like receptor activation that further promotes immune-mediated liver injury [18, 20, 21]. Standard treatment regimens with interferon (IFN)-α and nucleoside/nucleotide analogs are used for the treatment of CHB [22]. However, the exact role of gut microbiota equilibrium in the treatment of patients infected with HBV is still unknown. Animal studies have shown that the gut microbiota plays an important role in the initiation and development of CHB [23, 24]. Alternatively, during an HBV infection, bacteria from the *Leptospiraceae* family might play a role in the management of an HBV infection by reducing bacterial translocation and decreasing LPS content [25, 26]. Chou et al. reported that the gut microbiota plays a key role in the HBV-related immune response [27]. Recent studies have shown that entecavir administration could ameliorate HBV-induced disruption of the gut microbiota in humans [28]. However, there are no reports of changes in the intestinal microbiota after treatment with tenofovir dipivoxil (TDF). Therefore, in this study, we aimed to investigate the changes in the intestinal microbiota before and after treatment with TDF and the association with inflammatory factors.

## Methods

### Recruitment of subjects

The inclusion criteria for this study were hepatitis B virus (HBV)-infected patients with liver enzymes less than three times the upper limit of the normal range and without liver fibrosis. The inclusion criteria for this study were hepatitis B virus (HBV)-infected patients with liver enzymes less than three times the upper limit of the normal range and without liver fibrosis. Recognizing the potential for transient fluctuations in liver enzymes, we clarified our criteria to specify that patients with occasional ALT levels exceeding 150 U/L were not excluded, provided these spikes were transient and not indicative of ongoing liver damage or fibrosis.

Exclusion criteria were: alcoholism, presence of genetic or metabolic liver disease, comorbidity with hepatitis C virus, hepatitis D virus, human immunodeficiency virus, or cytomegalovirus; concurrent malignancies, autoimmune liver disease, cirrhosis, nonalcoholic steatohepatitis, pregnant or lactating women, concurrent medical conditions such as diabetes, heart failure, inflammatory bowel disease, irritable bowel syndrome, kidney damage, acute gastroenteritis 8 days prior to enrollment, patients with Parkinson’s disease, Alzheimer’s disease, stroke, mental illness; patients who have had infections, used antibiotics, or used probiotics within 3 months. Patients with a history of other antiviral therapies such as interferon, adefovir, or telbivudine were excluded.

### Detection of inflammatory factors

Serum samples from 48 No-NAs, 61 TDF, and 42 HC were collected for measurement of 20 human TH1-TH2-TH17 cytokines, including granulocyte-macrophage colony-stimulating factor (GM-CSF), interferon gamma (IFNγ), interleukin (IL)-1 beta, IL-2, IL-4, IL-5, IL-6, IL-10, IL-12p70, IL-13, IL-17 A, IL-17 F, IL-21, IL-22, IL-23, IL-28 A (IFN-lambda 2), macrophage inflammatory protein-3 alpha (MIP-3α), TGF beta 1, TNF alpha and TNF beta according to the manufacturer’s instructions (Quantibody ^®^ Human TH17 Array 1, Cat. No. QAH-TH17-1, RayBiotech). IL-18 was detected using a human IL-18 ELISA kit (cat no. EHC127, Neobioscience).

### Fecal samples collection and 16 S rRNA gene sequencing

Participant stool samples were collected and stored in a refrigerator at -80 °C before DNA extraction. Bacterial DNA was extracted according to the manufacturer’s instructions for DNA extraction using the E.Z.N.A.^®^ Stool DNA Kit (Omega Bio-tek, Inc., GA). PCR amplification of the bacterial 16 S rRNA gene V3-V4 region was performed using primers 341 F and 805R (341 F:5′-CCTACGGGNGGCWGCAG-3′; 805R:5′-GACTACHVGGTATCTAATCC-3′). PCR products from each sample were indexed and mixed in equal ratios for sequencing using the Miseq platform (Illumina Inc, USA) according to the manufacturer’s instructions.

### Bioinformatics analysis

Sequence read pairs were demultiplexed and reads were merged using USEARCH version 11.0. Sequences that could not be spliced and chimeras were both removed. Chimeras were eliminated using UCHIME software. Operational Taxonomy Units (OTUs) were clustered based on 97% similarity using UPARSE software [29] (version 7.1 http://drive5.com/uparse/) after chimeric sequences were removed. The phylogenetic affiliation of each representative 16 S rRNA gene sequence was annotated using the SILVA database (SSU138). Mothur v1.42.1 was used to calculate ACE, Chao1, Shannon and Simpson estimators of alpha diversity [30].

Principal coordinate analysis (PCoA) was performed using Bray-Curtis dissimilarity to visualize microbial communities. Permutational multivariate analysis of variance (PERMANOVA) was used to evaluate beta diversity using the adonis function of vegan [31]. Linear discriminant analysis (LDA) effect size (LEfSe) used the Kruskal-Wallis rank sum test in combination with LDA to detect traits with significantly different abundances at the genus and functional levels.

Predictive functional profiling was performed with the taxonomic profiles obtained from 16 S rRNA gene amplicon sequencing using Pipeline Phylogenetic Investigation of Communities by Reconstruction of Unobserved States (PICRUSt) version 2.4.1 [32]. LEfSe, based on the functional profiles predicted by PICRUSt, was used to predict the differences between the gut microbiota of the three groups.

Correlations between variables were calculated using Spearman’s rank correlation analysis of the R package Hmisc. Association p-values less than 0.05 were considered relevant.

### Statistics analysis

Differences between groups were assessed with the nonparametric Kruskal-Wallis and Mann-Whitney U tests. Relative abundance values for each bacterium were presented as mean or median. The chi-square test was used to compare categorical data. P values < 0.05 were considered statistically significant. Statistical analyzes were performed using R 4.1.

Bonferroni correction was applied to account for multiple testing in our statistical analyses.

The Shapiro-Wilk test was used to assess the normal distribution of our data.

## Results

### Patient characteristics

A total of 109 HBV-infected patients were recruited from Huashan Hospital, including 48 patients who were not taking nucleoside analogs (No-NAs group), 61 patients who had been continuously treated with oral TDF for more than 3 months (TDF group), and an additionally recruited 42 healthy subjects (HC group). For the TDF group, we divided them into two subgroups according to HBeAg antigen: Stage 1 were HBV e antigen (HBeAg)-negative patients and Stage 2 were HBeAg-positive (+) patients. HBeAg + patients were those with positive surface antigen, e-antigen, and core antibody, and HBeAg - patients were those with positive surface antigen, e-antigen, and core antibody and negative e-antigen. We also investigated the difference in gut microbiota between HBeAg-positive and negative hepatitis B patients treated with TDF. In addition, we examined the effects duration of TDF administration had on gut microbiota. Age, sex, and body mass index (BMI) of healthy controls were not significantly different from those of HBV-positive individuals (Table S1).

### Clinical characteristics

After TDF treatment, CHB patients showed a significant reduction in HBV DNA burden (*p* < 0.001) (Figure S1). In addition, r-glutamyltransferase, alanine aminotransferase and aspartate aminotransferase levels were significantly decreased in the TDF group compared to the No-NAs group, while prealbumin levels were significantly increased (Table S2).

### Compositional analysis of gut microbiota

**Fig. 1:**
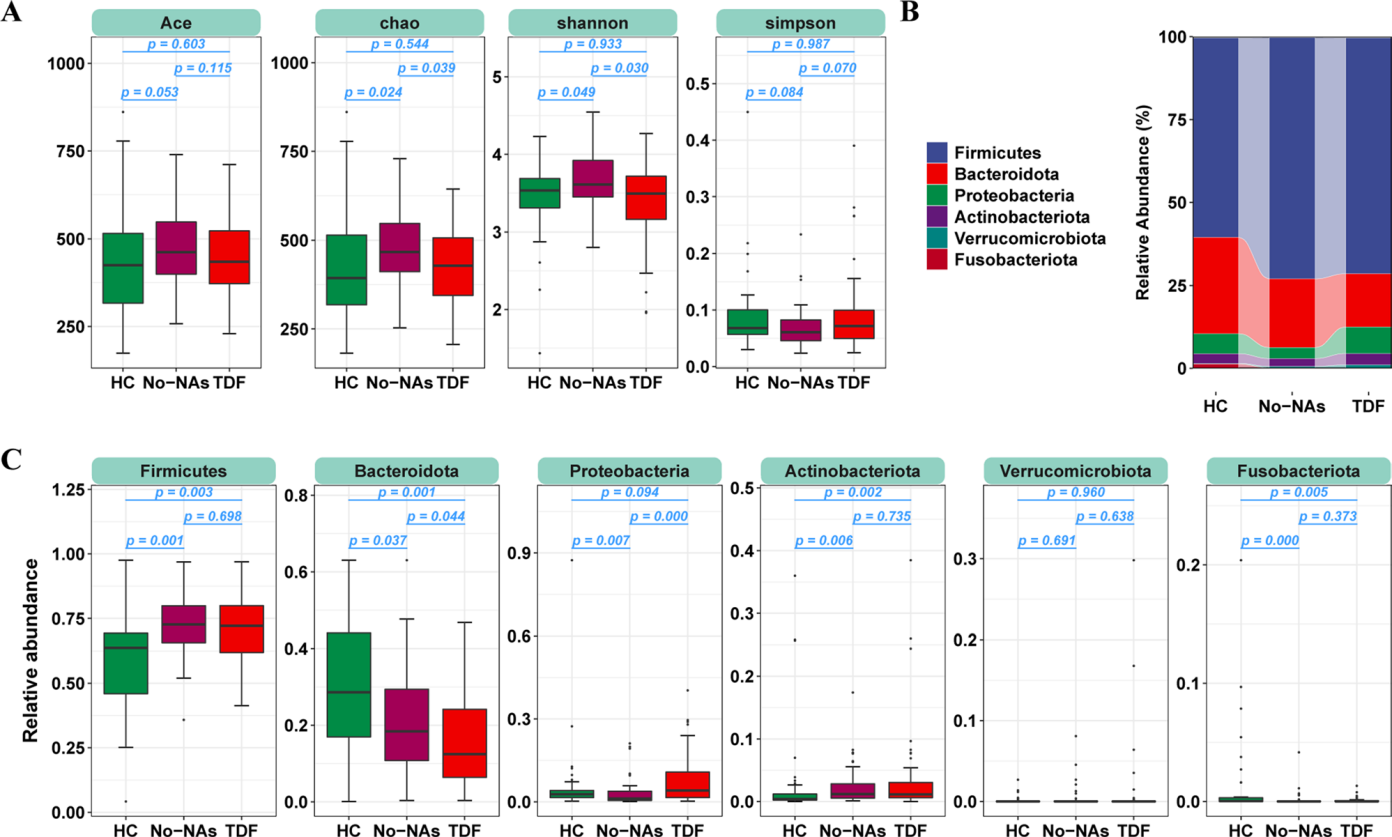
Effect of TDF on intestinal flora in patients with CHB. (**A**) Effect of TDF on alpha diversity of intestinal flora. (**B**, **C**) Relative abundances of each bacterial phylum in the patients and healthy controls

The relative abundances of each bacterial phylum in the patients and healthy controls are shown in Fig. 1B. In the three groups, the major phyla were *Firmicutes*, *Bacteroidota*, *Proteobacteria*, *Actinobacteria*, *Verrucomicrobiota*, and *Fusobacteriota*, of which *Firmicutes*, *Bacteroidetes*, *Proteobacteria*, and *Actinobacteria* accounted for more than 98% (Fig. 1B). The relative abundance of *Bacteroidota* gradually decreased from the HC group to the No-NAs and TDF groups. *Firmicutes* and *Actinobacteria* were more abundant in the No-NAs and TDF groups than in the HC group. The relative abundance of *Fusobacteriota* was significantly higher in the No-NAs group than in the HC group. In addition, the relative abundance of *Proteobacteria* was significantly lower in the No-NAs group than in the HC and TDF groups (Fig. 1C).

### Analysis of gut microbiota differences at the genus level among the three groups

**Fig. 2:**
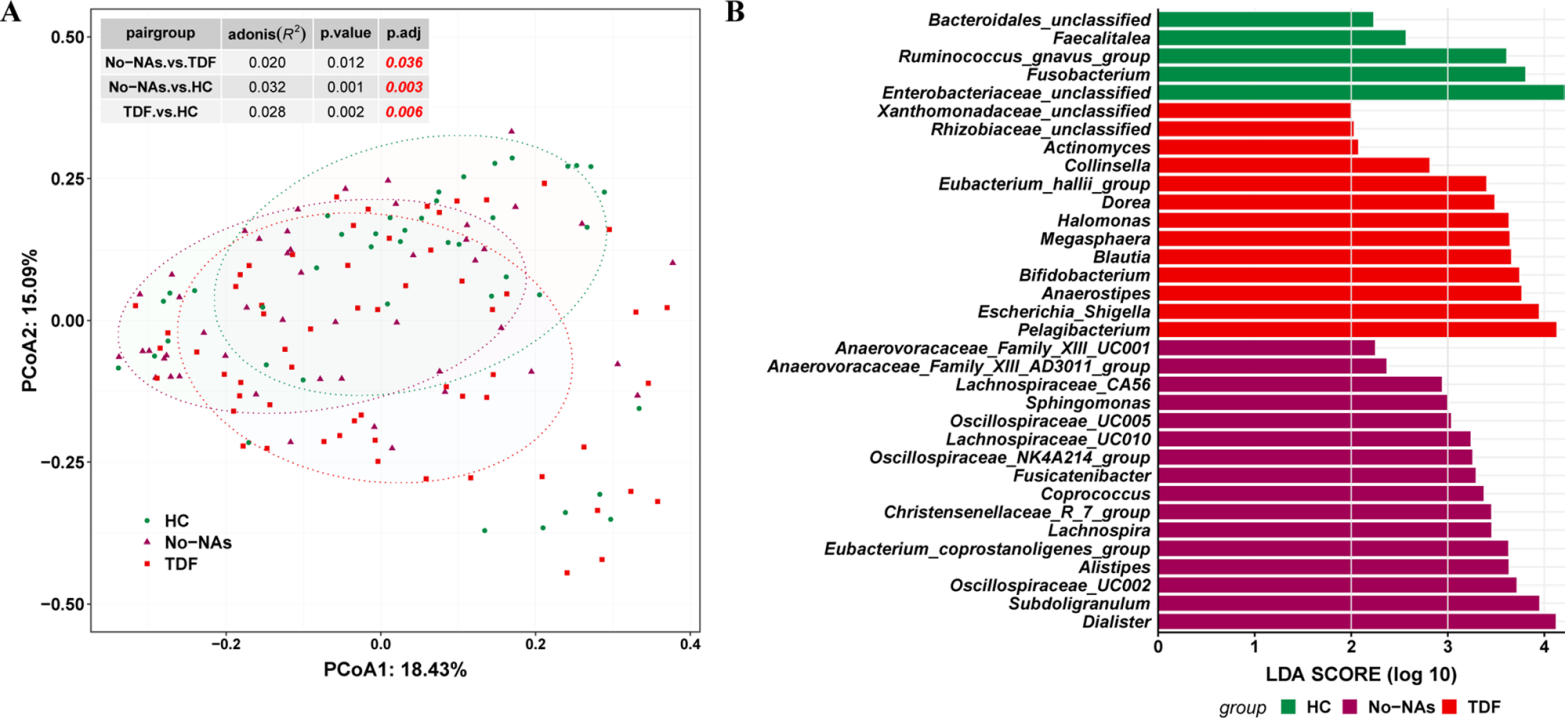
Gut microbiota differences at the genus level among the three groups. (**A**) Principal coordinate analysis of β diversity of flora based on Bary-Curits distance (PCoA). (**B**) LEfSe analysis at genus level

PCoA based on the Bray-Curtis distance matrix showed clear differentiation of bacterial communities among HC, No-NAs, and TDF groups (Fig. 2A). PERMANOVA pairwise interactions were used to identify significant differences between groups (*p* < 0.05, table in Fig. 2A). Data on the mean relative abundances of each bacterial genus in patients and healthy controls are shown in Table S3. We used LEfSe analysis to identify the genera that were responsible for the differences in fecal microbiota among the three groups. The differences in the intestinal microbiota of the three groups are shown in Fig. 2B. Some beneficial bacteria such as the *Ruminococcus_gnavus_group* and *Fusobacterium* were enriched in the HC group, whereas some opportunistic pathogens such as *Sphingomonas* and *Escherichia/Shigella* were increased in the No-NAs and TDF groups.

### Identification of differential genera and key taxa

**Fig. 3:**
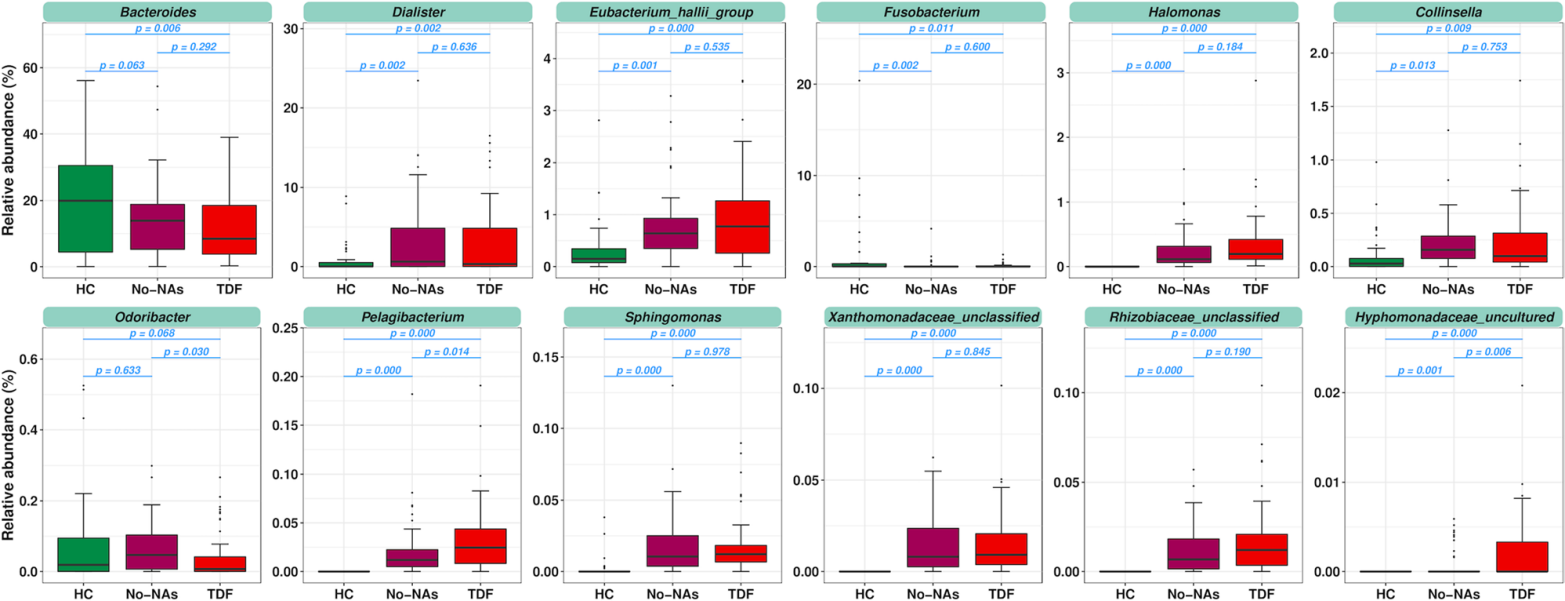
Relative abundances of differential genera and potential key taxa responsible for the differences in HC, No-NAs, and TDF groups

To identify the key bacteria responsible for the differences in HC, No-NAs, and TDF groups, a taxonomy-based bacterial comparison was performed (Fig. 3). Abundances of Bacteroides were significantly decreased in the TDF groups. Abundances of Fusobacterium were significantly decreased in the No-NAs and TDF groups, but there was no significant difference between the No-NAs and TDF groups. Among the various predominant genera, *Dialister*, *Eubacterium_hallii_group*, *Halomonas*, *Collinsella*, *Sphingomonas*, *Xanthomonadaceae_unclassified*, and *Rhizobiaceae_unclassified* were overrepresented in the No-NAs and TDF groups, but there was no significant difference between the No-NAs and TDF groups. In addition, the abundance of *Pelagibacterium* and *Hyphomonadaceae_uncultured* gradually increased in the HC, No-NAs, and TDF groups. In particular, the abundance of *Odoribacter* was significantly reduced in the TDF group, while there was no significant difference between the HC and No-NAs groups (Fig. 3).

### Comparison of inflammation profiles

The transfer of lipopolysaccharide (LPS) from the intestine to the blood can cause immune activation. Normally, serum inflammatory factors are elevated in CHB patients. Therefore, our study focused on the effect of TDF treatment on serum inflammatory factors. Among the 21 inflammatory factors (IL -18, GM-CSF, IFNγ, IL -1b, IL -2, IL -4, IL -5, IL -6, IL -10, IL -12p70, IL -13, IL -17, IL -17 F, IL -21, IL -22, IL -23, IL -28 A, MIP-3α, TGFβ1, TNFα, TNFβ) that were examined, there was no significant difference in marker levels between the TDF and No-NAs groups (Table S4).

### Correlation between gut microbiota and inflammatory factors

**Fig. 4:**
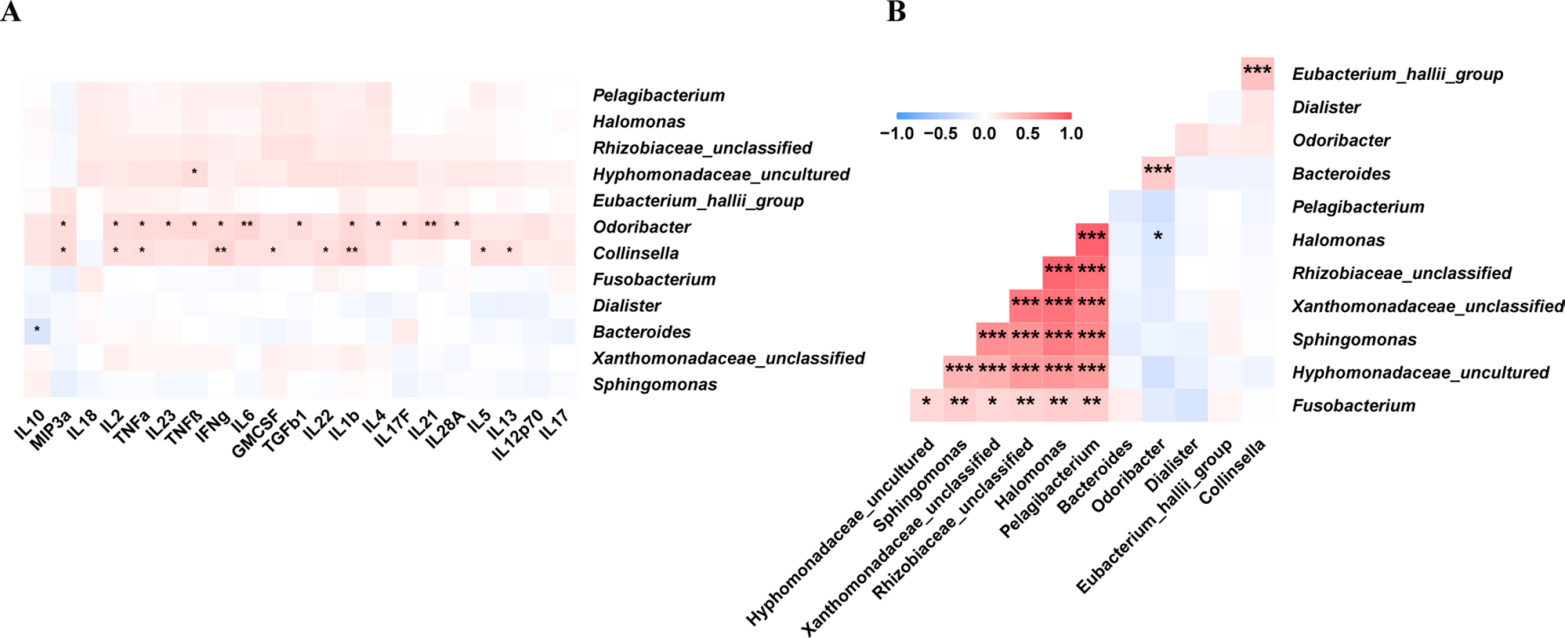
Correlation between clinical parameters and gut microbiota (**A**) Correlation between inflammatory factors and gut microbiota. (**B**) Correlation between microbial genera enriched in the No-NAs and TDF groups. *p-value smaller than 0.05, **p-value smaller than 0.01 and ***p-value smaller than 0.001

Spearman correlations between the relative abundance of bacterial genera and the levels of inflammatory factors were evaluated (Figure S2). Inflammatory factors were positively correlated with opportunistic pathogens, such as *Escherichia/Shigella*, while negatively correlated with some beneficial bacteria, such as *Stomatobaculum* and *Atopobium*. We focused on the correlation between the three groups of significantly different genera (Fig. 4A) and inflammatory factors. We found a positive correlation between *Odoribacter* and inflammatory factors. Alternatively, there was a significant correlation between microbial genera enriched in the No-NAs and TDF groups (Fig. 4B).

### HBV specific trends in functional profiles

**Fig. 5:**
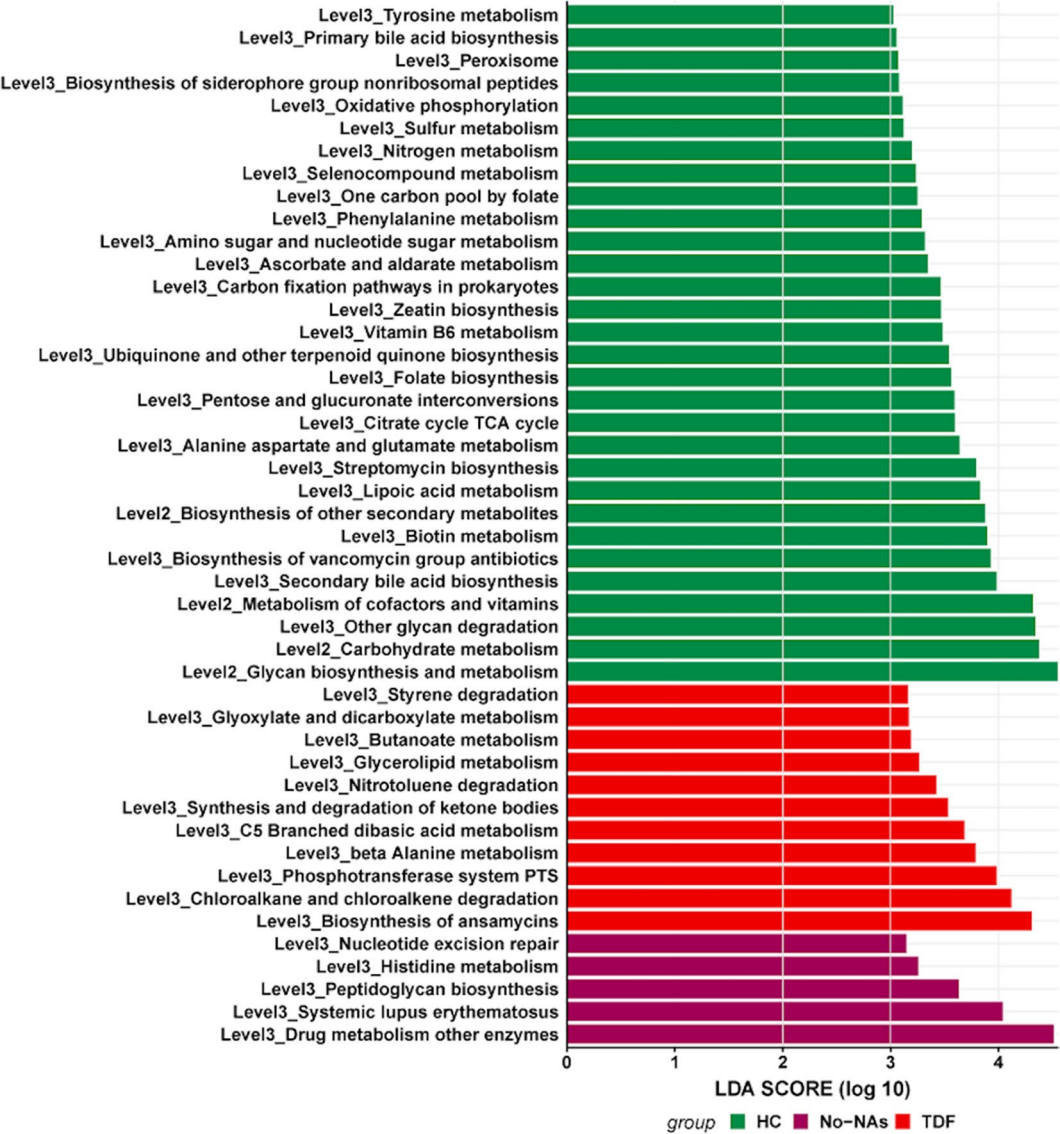
KEGG pathway analysis of HC, No-NAs, and TDF groups

To investigate the HBV-specific functional properties of the current microbiome dataset, we sought to examine HBV-specific trends in the functional composition of the gut microbiota in the three groups. We observed an effect of HBV and TDF treatment on the functional profiles of the gut microbiota. As shown in Fig. 5, KEGG pathway analysis revealed that 46 pathways related to metabolism and disease were significantly enriched in the three groups. Based on LDA selection, 30 predicted microbial functions, including metabolism of cofactors and vitamins, biosynthesis of secondary bile acids, biotin metabolism, folate biosynthesis, vitamin B6 metabolism, and zeatin biosynthesis were remarkably enriched in the HC group; 5 functions, including drug metabolism and other enzymes, were remarkably enriched in the No-NAs group; and 11 predicted microbial functions, including butanoate metabolism and styrene degradation, were remarkably enriched in the TDF group (*p* < 0.05). The observed differences in gut microbiota between the TDF-treated and No-NA cohorts may be influenced by the varying levels of hepatic inflammation, as indicated by significantly higher AST and ALT levels in the No-NA cohort. Hepatic inflammation and liver injury can affect gut microbiota through altered bile acid metabolism, increased intestinal permeability, and systemic inflammatory responses. Consequently, the gut microbiota changes observed may not be solely attributable to TDF treatment but could also result from reduced hepatic inflammation in the TDF-treated group. Future studies should stratify patients based on liver enzyme levels and conduct detailed analyses of bile acid profiles and inflammatory markers, as well as longitudinally track gut microbiota changes before and after TDF treatment to better understand these relationships.

### Differential analysis between two phases of the TDF group

On the day of specimen collection, we grouped patients treated with TDF according to their e-antigen status, namely: Phase 1 being the HBV e-antigen (HBeAg)-negative patient group, and Phase 2 being the HBeAg-positive patient group. Phase 1 and Phase 2 clinical baseline tables were shown in Table S5. The prealbumin of Phase 2 was significantly higher than that of Phase 1. In addition, there was no significant difference in inflammatory factors between Phase 1 and Phase 2, except IL4 (*p* = 0.047), which was significantly lower within Phase 2 (Table S6).

**Fig. 6:**
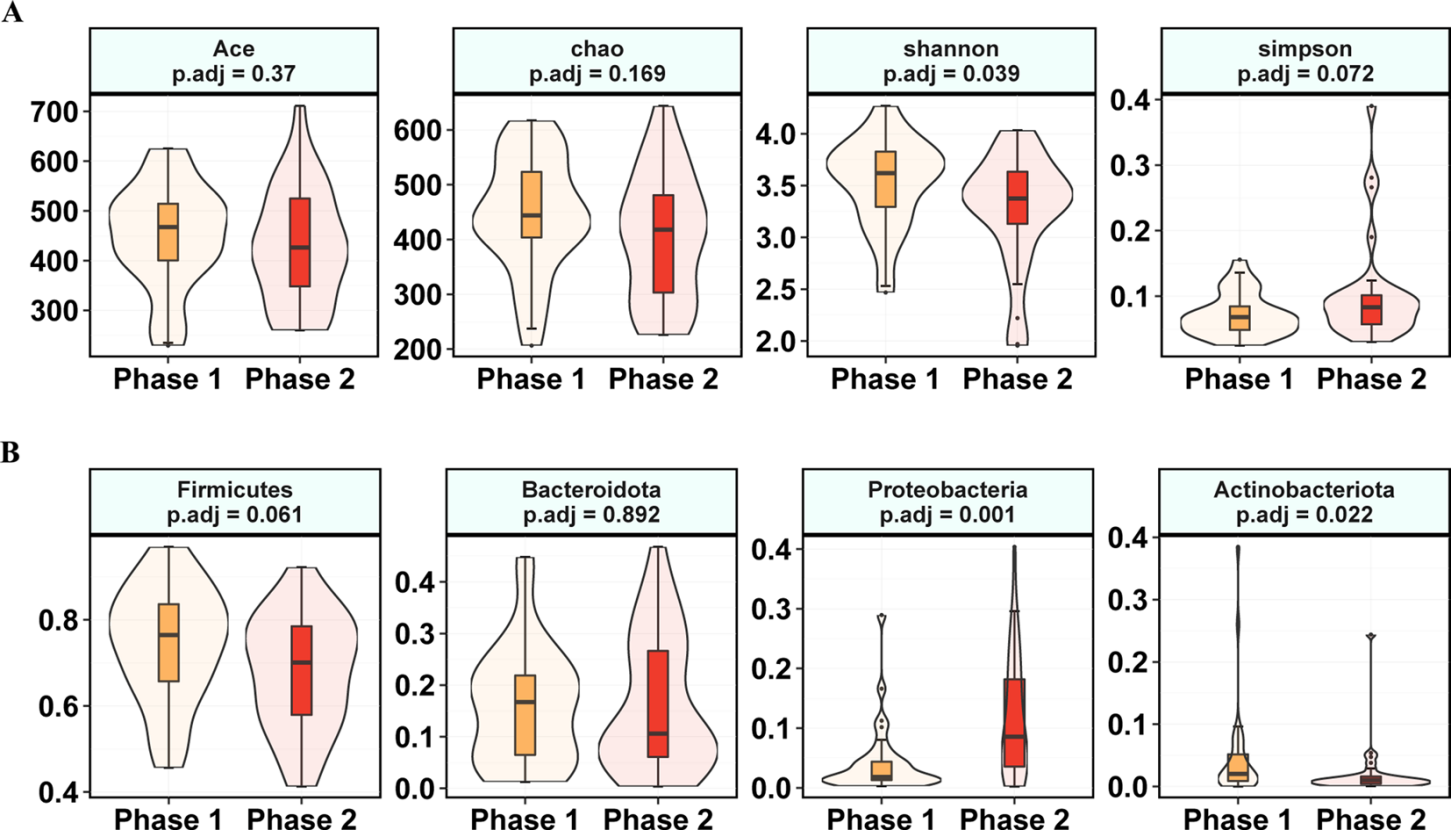
Effect of TDF on intestinal flora in patients with CHB. (**A**) Effect of TDF on alpha diversity of intestinal flora. (**B**) Phylum level differences of microbiota. Phase 1 are HBeAg-negative patients and phase 2 are HBeAg-positive patients

There was no significant difference in the richness of gut microbiota in the two subgroups, and it was found that the diversity of intestinal microbiota in Phase 2 was significantly reduced (reflected in the Shannon index, but there was no significant difference in the Simpson index) (Fig. 6A). In addition, at phylum level *Firmicutes* and *Bacteroidota* were not significantly different. *Proteobacteria* were significantly increased in Phase 2 compared with Phase 1, while *Actinobacteriota* was significantly decreased (Fig. 6B).

**Fig. 7:**
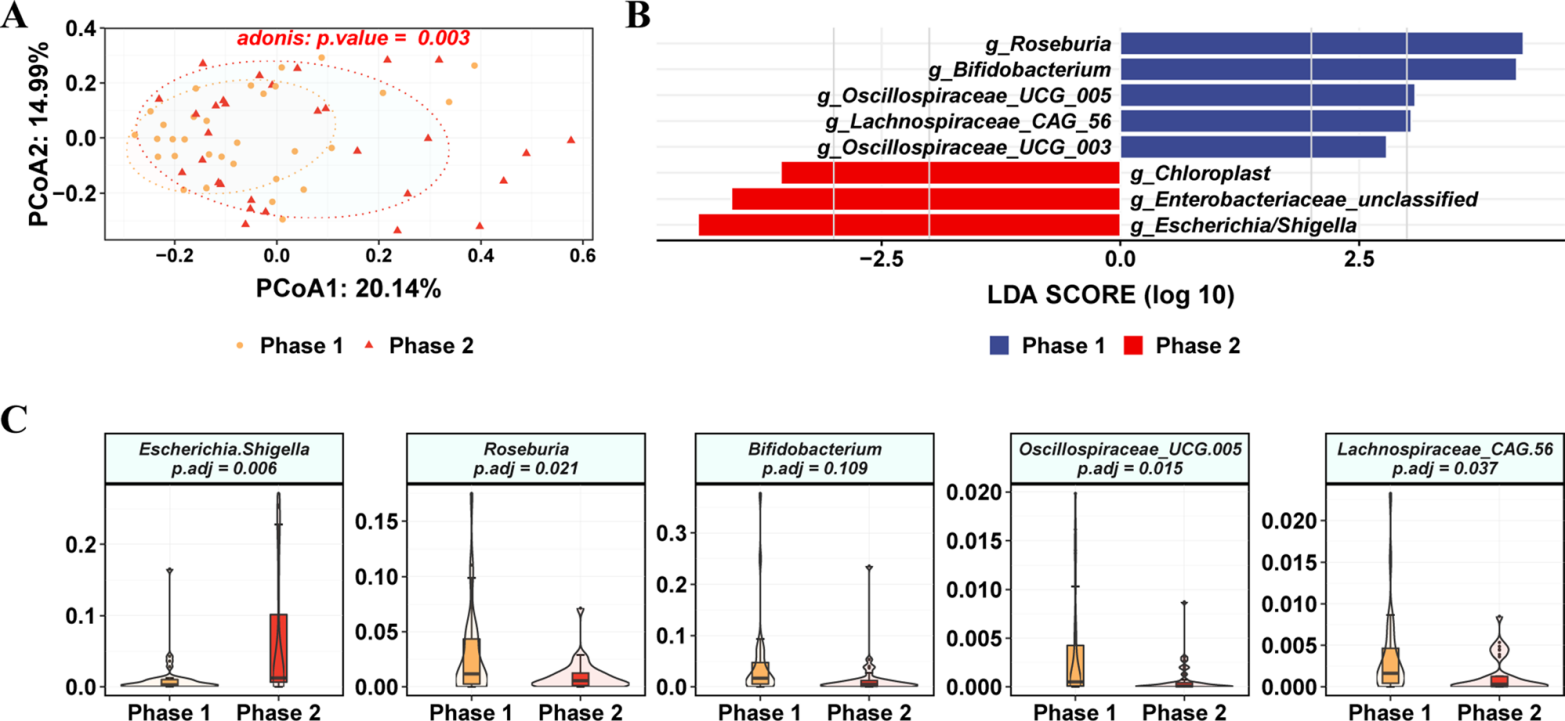
Characteristics of gut microbiota in patients with hepatitis B and differences in intestinal flora. (**A**) Principal coordinate analysis of β diversity of flora based on Bary-Curits distance (PCoA). (**B**) LEfSe analysis at genus level. (**C**) Relative abundances at the genus level. Phase 1 are HBeAg-negative patients and phase 2 are HBeAg-positive patients

The differences between Phase 1 and Phase 2 were also manifested at the genus level. PCoA results showed that there was a significant difference between the two subgroups (adonis *p* value = 0.003) (Fig. 7A). Some beneficial bacteria such as *Roseburia* and *Bifidobacterium* were found to be enriched in the Phase 1 group, while some opportunistic pathogens such as *Escherichia/Shigella* were enriched in the Phase 2 group (Fig. 7B). Further analysis revealed that *Escherichia/Shigella* was significantly increased in Phase 2, while the relative abundance of *Roseburia*, *Oscillospiraceae_UCG.005* and Lachnospiraceae*_CAG.56* was significantly decreased in Phase 2 (Fig. 7C). No significant change in the relative abundance of *Bifidobacterium* was observed in these two subgroups.

### Effect of duration of TDF administration on gut microbiota

To explore the effect of medication time on gut microbiota, we divided the TDF group into two groups according to medication time less than 24 months (Short-term group) and more than 24 months (Long-term group). Beta diversity analysis revealed that there was a significant difference between the two groups (Figure S3). At the genus level, we found that the relative abundance of *Faecalibacterium* increased with increasing medication time, while *Dialister* was conversely decreased (Table S7).

## Discussion

Recently, researchers have begun to investigate the relationship between HBV infection and the gut microbiota. Infection with HBV resulted in changes in the relative abundance of beneficial and opportunistic bacteria compared to healthy individuals [1, 33–35]. Currently, antiviral drugs (such as tenofovir, entecavir, and tenofovir alafenamide) are the most effective oral drugs for combating HBV [36–38]. The main mechanism of these nucleoside analogs is to block HBV replication, but whether these drugs can directly affect the gut microbiota is still unknown. Our results suggest that TDF treatment can significantly reduce the viral load in CHB patients, as well as induce significant changes in gut microbiota. We observed differences in the abundance of *Firmicutes* and *Bacteroidota* between the No-NAs and TDF groups. Chen et al. have also found that a change in *Bacteroidota* is characteristic of CHB or alcohol-induced cirrhosis [9]. However, our results suggest that TDF treatment was unable to fully restore the gut microbiota equilibrium to healthy control levels, nor were the levels of inflammatory factors able to improve significantly. This is particularly important because, within the predictive, preventive, and personalized medicine framework, the role of systemic inflammation as a health-related communication tool between the human host and the gut microbiota cannot be overlooked [39]. The interplay between the host and the microbiota may influence the severity of hepatitis infection and the therapeutic outcomes, underscoring the need to account for the host’s inflammatory status and gut microbiota modulation in treatment strategies. Nucleoside analogs (such as TDF) are effective in reducing HBV load [38], and in most cases able to prevent liver disease progression [40]; however, a better understanding of the complex interactions between HBV and gut bacteria is essential to determine whether HBV treatment strategies should target the virus, host gut bacteria, or a combination of both in order to achieve functional therapy.

Our analysis has extended to examine the influence of the duration of TDF therapy on the gut microbiota among CHB patients. Notably, an extended period of TDF administration is correlated with substantial enhancements in the gut microbiota composition, suggesting a time-dependent beneficial effect. The observed improvements potentially stem from a sustained suppression of HBV replication, which in turn lessens systemic inflammation and viral burden. Additionally, enhanced liver function, possibly a consequence of TDF treatment, could exert a positive influence on the gut microbiota through the gut-liver axis. Furthermore, the stabilization of immune responses may contribute to a reduction in chronic inflammation, fostering a more balanced gut ecosystem. The exact nature of TDF’s impact on the gut microbiota, whether direct or indirect, merits further investigation.

The results of this study indicate that *Proteobacteria* were more abundant in HBV-infected individuals, which is consistent with other studies [33, 41]. During HBV infection, a decrease in *Bacteroidota* and an increase in *Proteobacteria* were observed [12]. *Fusobacterium* is a major member of the phylum *Fusobacteria*, which have been reported to be associated with intestinal inflammation [42, 43]. However, the present study found a decreased abundance of *Fusobacterium* in the No-NAs and TDF groups, which is inconsistent with the reported results [26, 44]. Additionally, no significant correlation was found between inflammatory factors and *Fusobacterium* in the present study (Fig. 4A). The phylum *Bacteroidota* (especially the genus *Bacteroides*), which have anti-inflammatory characteristics [45, 46], was depleted in the No-NAs and TDF groups. This is consistent with the results reported in the literature [26, 47–50]. *Actinobacteria* was increased in the No-NAs and TDF groups, and a similar trend has been found [48]. Furthermore, *Bifidobacterium* (the phylum of *Actinobacteria*) was not significantly different between the No-NAs and TDF groups, nor in the subgroups of the TDF group (Fig. 7C).

Moreover, we found a significant difference in gut microbiota between Phases 1 and 2 in the TDF group, suggesting that the elimination of HBV DNA could potentially cause changes in gut microbiota. It was also reported that the use of fecal microbiota transplantation in the reconstruction of the gut microbiota promoted the clearance of HBeAg in CHB patients [51]. Further investigation revealed that the duration of TDF medication also had a potential effect on intestinal bacteria (Figure S3). As medication time increased, *Faecalibacterium* populations tended to increase, while *Dialister* populations tended to decrease (Table S7). However, the detailed mechanisms by which the gut microbiota regulate intrahepatic anti-HBV immunity remain largely unknown.

Crucially, there are a large number of functional genes derived from gut microbiota that are found in healthy controls [52, 53]. Gut bacteria can digest and absorb ingested complex carbohydrates from the diet that cannot be digested by human enzymes and in doing so are able to synthesize some essential substances for the host [54–56]. It has also been reported that the abundance of functional genes that are important for nutrient metabolism, including amino acid, nucleotide, and lipid metabolism; and isoprenoid biosynthesis are significantly reduced in HBV-related cirrhosis compared with healthy controls [57]. Our results were consistent with the reported literature, with vitamin synthesis being significantly decreased in the No-NAs and TDF groups compared to the HC group, while some were increased with drug metabolism (Fig. 5). Usually, CHB patients have altered intestinal permeability, increased bacterial translocation and portal vein endotoxin load, which leads to Toll-like receptor activation in the liver and promotes immune-mediated liver injury [12, 20]. At the phylum level, we observed a trend towards increased levels of *Proteobacteria* in the TDF group (Fig. 1C). At the genus level, some genera belonging to *Proteobacteria* such as *Halomonas*, *Sphingomonas*, *Xanthomonadaceae*_*unclassified* and *Rhizobiaceae_unclassified* were overrepresented in the No-NAs and TDF groups (Fig. 3). LPS is a major component of the outer membrane of Gram-negative bacteria and a potent ligand for the host receptor toll-like receptor 4 (TLR4), which plays an important role in sensing bacteria [58]. Chou et al. reported that the LPS-TLR4 signaling pathway plays an important role in immune system tolerance to HBV infection [27]. Our study showed that although TDF therapy was able to reduce HBV load (Figure S1), it did not effectively improve inflammatory factor levels. Generally, alterations in the gut microbiota and colonization by opportunistic pathogens increase the risk of comorbidity in CHB patients. At present, a new therapeutic target has been established through the study of the gut microbiota [17]. HBV infection can increase intestinal permeability, damage the intestinal barrier, facilitate bacterial overgrowth and bacterial translocation, and promote immune-mediated liver injury [59–61]. Therefore, gut microbiota play an important role in host health, and intervention of patients’ gut microbiomes may provide good support in the curing of chronic HBV infections.

In light of our previous study on the effects of tenofovir alafenamide (TAF) on gut microbiota in CHB patients, it is important to compare those findings with our current results on tenofovir disoproxil fumarate (TDF). Both treatments are associated with overall improvements in gut microbiota profiles, including increased microbial diversity and reduced pathobionts. However, specific bacterial taxa affected differed between TDF and TAF, indicating distinct impacts despite similar antiviral mechanisms. Additionally, the time-dependent effects on gut microbiota were more pronounced with TDF. These differences could influence personalized treatment approaches, suggesting that certain patients might benefit more from one treatment over the other based on their gut microbiota profiles and health status. Future research should focus on direct comparisons of TDF and TAF in a single cohort, the effects of switching between treatments, and the functional aspects of gut microbiota changes.

Taken together, these results suggest that inflammatory factors are involved in the observed dysbiosis of the gut microbiota in HBV-infected patients. However, the No-NAs and TDF groups were not comprised of the same individuals before and after treatment, meaning between-person confounders cannot be ruled out. Therefore, future self-controlled studies are needed to investigate the changes in gut microbiota in hepatitis B patients before and after taking TDF to explore the effect of TDF on gut microbiota. This study cannot directly answer the question of whether the changes in microbiota are pathogenic or the result of systemic HBV-related inflammatory factors. In this study, it was acknowledged that the different nutritional status and lifestyle habits of the subjects may have contributed to a bias in the analysis. On the other hand, although correlational analysis helps in linking inflammatory factors to the effects of dysbiosis, there is no direct manipulation of the microbiome to examine its relationship to disease in vitro or in vivo. Because of these limitations, well-designed prospective studies should be conducted in the future to confirm our findings. In addition, the exact mechanism of how HBV infection leads to gut dysbiosis requires further investigation. Additionally, while we observed associations between TDF treatment and alterations in gut microbiota composition in CHB patients, these findings should be interpreted with caution. The cross-sectional design of our study limits our ability to determine if these differences are directly caused by TDF treatment or influenced by other factors, such as improved liver function, duration of CHB, or lifestyle factors. To better understand the causal relationship, future research should include longitudinal studies to track microbiota changes over time, randomized controlled trials to compare TDF with placebo or alternative treatments, and mechanistic studies to explore TDF’s direct effects on gut microbiota. Our study provides valuable insights and lays the groundwork for further research to clarify the mechanisms and clinical implications of these associations.

## Conclusions

This study investigated the resulting changes of intestinal microbiota in HBV-infected patients and concluded that HBV causes dysregulation of intestinal microbiota. In addition, treatment with TDF was found to not completely restore the dysregulated intestinal microbiota. These findings suggest a potential influence on gut microbiota composition. On the other hand, TDF treatment also did not significantly improve serum inflammatory factor levels. Since disruption of the gut microbiota is associated with an increase in inflammatory factors, the therapeutic effect of TDF could be supplemented by also regulating gut microbiota. However, the available data in this field is still limited. In the future, a randomized controlled trial is needed to further investigate the changes in gut microbiota in HBV-infected patients not taking antiviral drugs (immune tolerance phase) and during medication. Such a randomized and self-controlled study would be helpful in investigating the changes in gut microbiota during the development of HBV and the role of gut microbiota play in the process of antiviral treatments.

## Electronic supplementary material

Below is the link to the electronic supplementary material.

Supplementary Material 1

Supplementary Material 2

Supplementary Material 3

Supplementary Material 4

Supplementary Material 5

Supplementary Material 6

Supplementary Material 7

Supplementary Material 8

## Data Availability

Raw sequencing data from both CHB patients and healthy participants are available in the NCBI Sequence Read Archive repository under accession numbers PRJNA778613 and PRJNA687563 respectively. The clinical metadata generated and analyzed during this study are included in this published article and its supplementary information files.
