# Supplementary material for: Role of tenofovir dipivoxil in gut microbiota recovery from HBV-infection induced dysbiosis
Source: BMC Microbiol. 2024 Sep 20;24:359. doi: 10.1186/s12866-024-03457-4 (PMC11414042; doi:10.1186/s12866-024-03457-4)
Supplement: Supplementary file 8 — Supplementary Material 8 [file 12866_2024_3457_MOESM8_ESM.docx]

**Supplementary Table Legends**

**Supplementary Table 3.** Comparison of gut microbiota at the genus level between HC, No-NAs, and TDF groups.

**Supplementary Table 4.** Comparison of serum inflammatory factors between No-NAs and TDF groups.

**Supplementary Table 5.** Demographic and clinical characteristics of Phase 1 and Phase 2 subgroups of TDF group.

**Supplementary Table 6.** Comparison of serum inflammatory factors of Phase 1 and Phase 2 subgroups.

**Supplementary Figures**


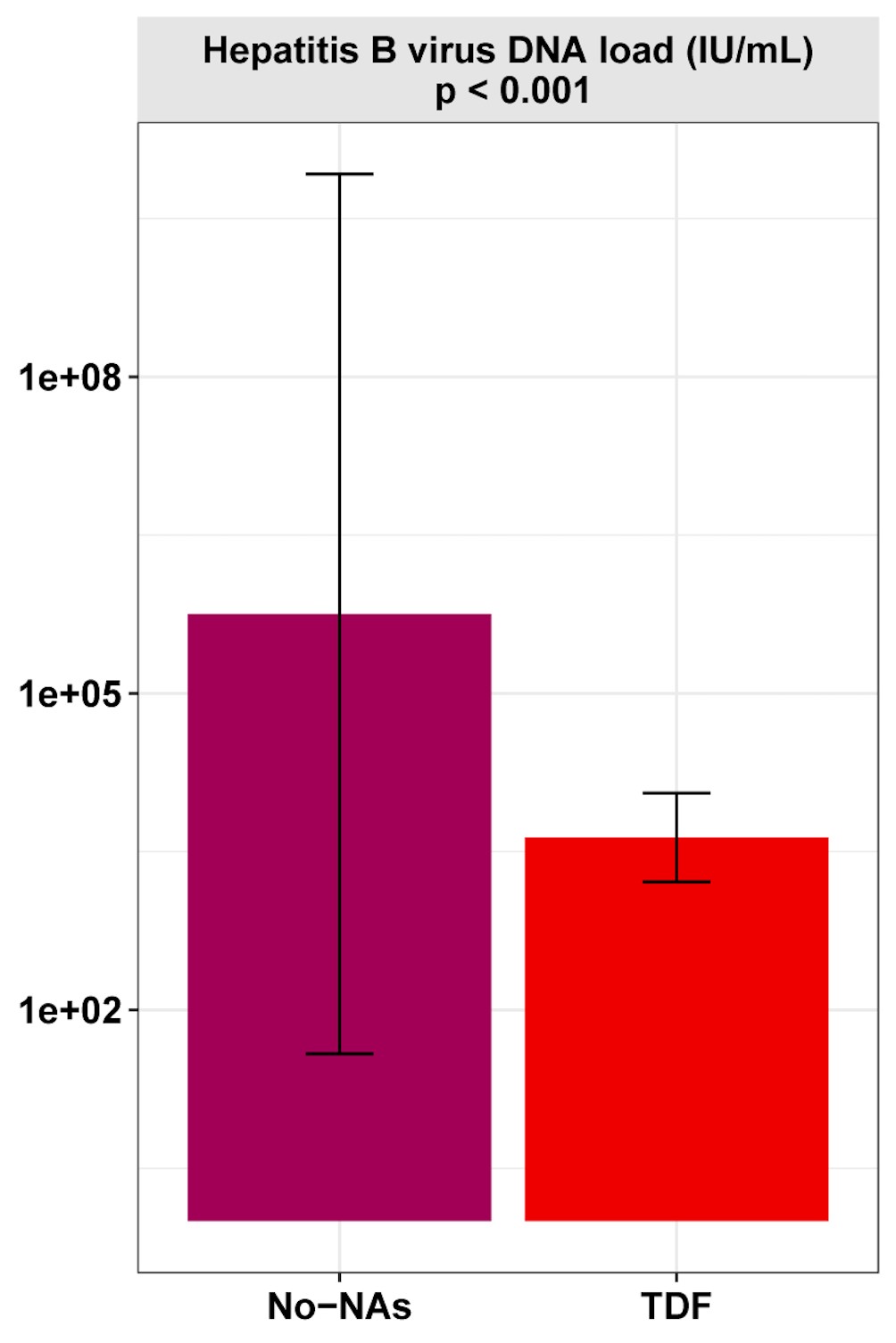


Figure S1. TDF effect on HBV viral load.


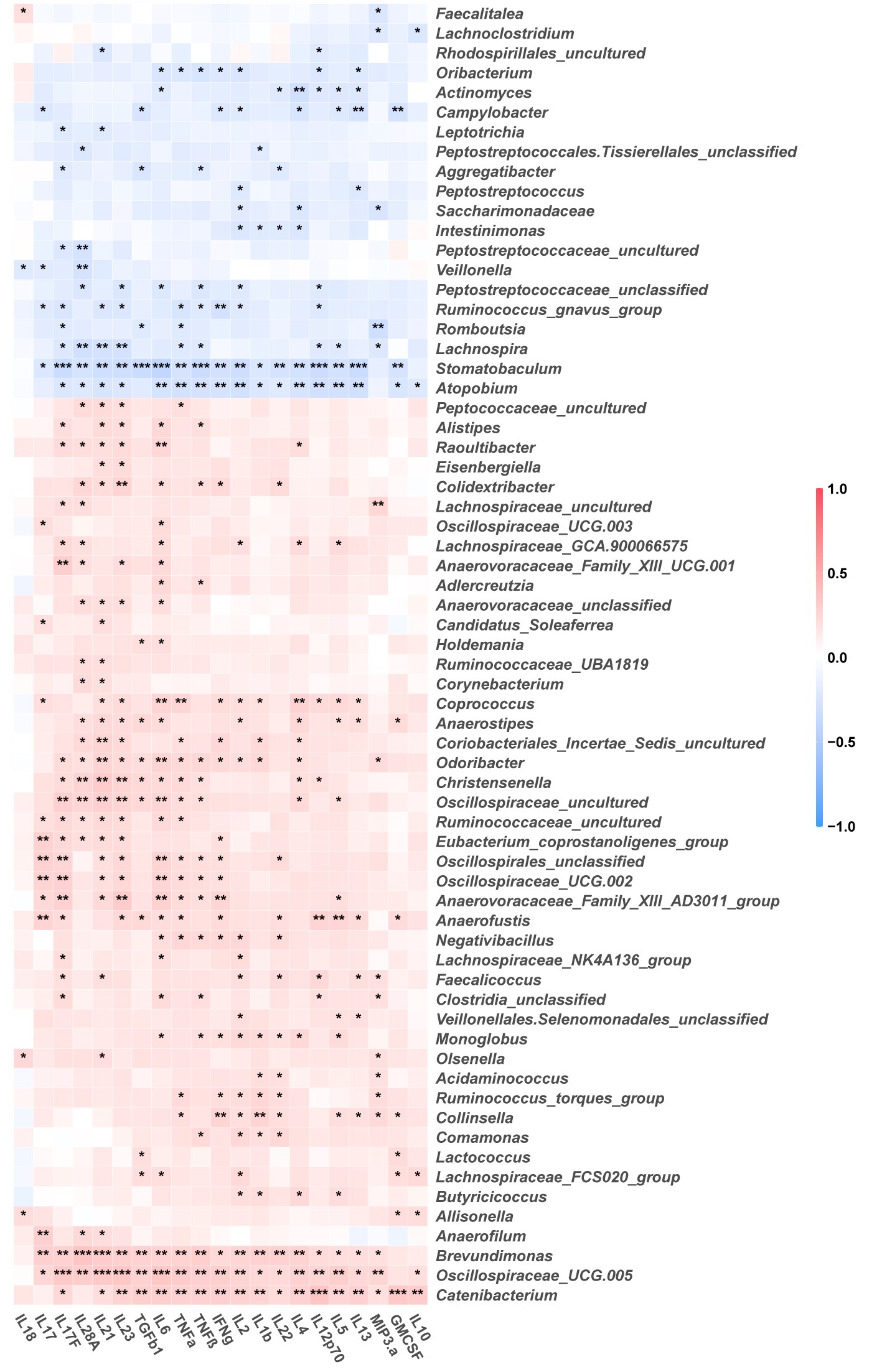


Figure S2. Spearman correlations between the relative abundance of bacterial genera and the levels of inflammatory factors.


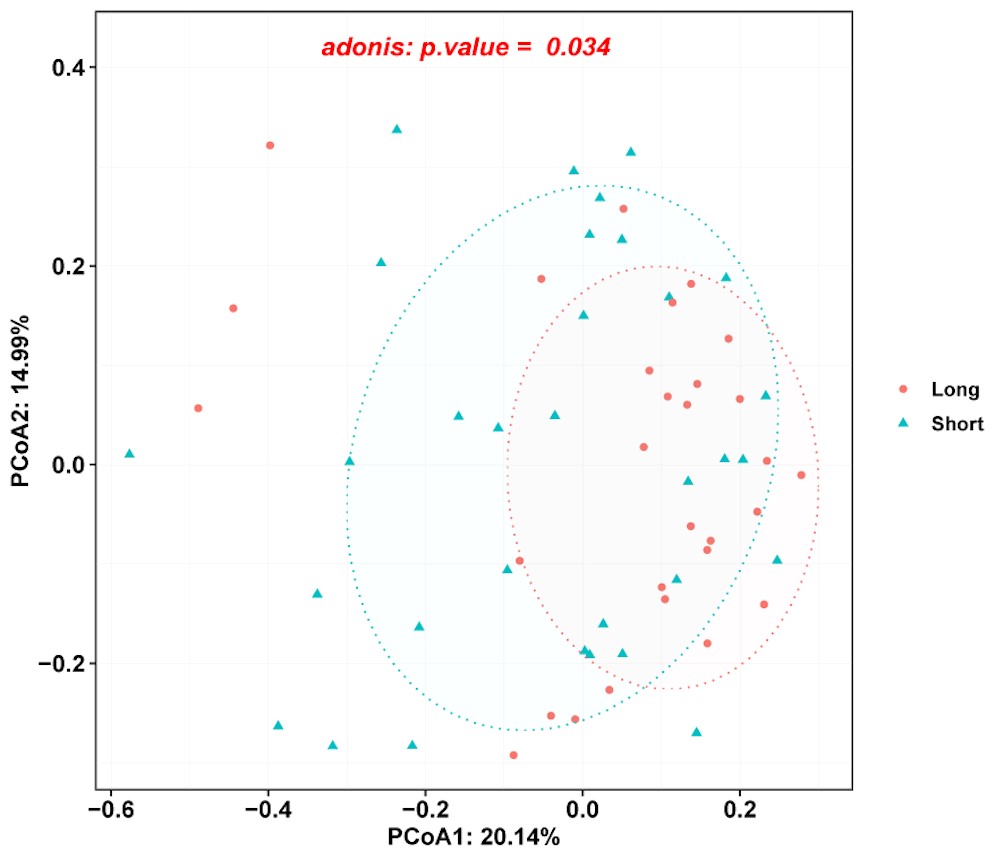


Figure S3. Principal coordinate analysis of β diversity of flora based on Bary-Curits distance (PCoA).
